# Dr. Hall, on Burns and Scalds

**Published:** 1807-02

**Authors:** R. Hall

**Affiliations:** Clement's Inn


### *To the Editors of the Medical and Physical Journal.*

GENTLEMEN,

AMONG all the causes that have operated to retard the progress of the healing art, and to defeat attempts towards experience and observation, none has, in our opinion, proved more injurious than the pride and spirit of system; for with the prejudices they engender, the most acute investigator may easily deceive himself, and be unwarily led to embrace error instead of truth. That the advocates for the use of terebinthinate applications, in cases of scalds and burns, have been influenced by such a spirit, we think must be allowed by any one who carefully peruses the different papers, inserted in the Medical and Physical Journal, on the comparative merit of the stimulant and refrigerant modes of treatment in such cases.* It appears to us

---

* Medical records sufficiently attest that the former of these modes of treatment is not so novel as some practitioners appear inclined to suppose. For though Heister, as far as we recollect, is the first who applied spirits of turpentine in burns and scalds, yet the use of external stimulants, under various modifications, was long before his time proposed by different authors. Among others, Sydenham recommended spirituous applications in such cases; a practice, which, it should seem, he was led to adopt from the notion that their employment tended to defend the subjacent skin from putrefaction. Boerhaave, who appears to have entertained similar sentiments respecting the antiseptic qualities of such remedies, was likewise an advocate for this method of treatment.

us that Dr. Kentish, in particular, has, in various instances, been led from an over-weening attachment to a favourite hypothesis, to extol and over-rate the benefits derived from the former of these modes of treatment, and to over-look, and depreciate, below their due value, the beneficial effects of the latter.

As to the stimulant plan, we ourselves cannot speak from our own experience, having never witnessed any cases in which it was employed; but in our hands topical cold has uniformly proved a prompt, easy, and efficacious mode of relieving such accidents; nor did we ever observe any of those injurious effects attributed by some to the operation of this remedy. On the contrary, it always appeared to us not only to obviate the symptomatic fever, but likewise in a great measure, when early and perseveringly employed, to prevent the serous exudations, and other circumstances, the necessary sequelæ of such injuries, when pain and inflammation are not speedily subdued. We are fully aware, however, that the merit of these two opposite modes of practice can only be justly appreciated by a careful comparison of experiments, instituted under circumstances as nearly similar as possible, by individuals capable of observation, and who have not prejudged the question at issue. For our own part, after a careful review of the whole evidence already before the public, we cannot clearly perceive, that the cures performed in this way were either less tedious, or the ease afforded to the patient more prompt and sudden, than what we ourselves, as well as others, have frequently witnessed in equally extensive injuries treated by topical cold. If any additional testimony can be necessary to confirm the salutary influence of this mode of treatment in remedying such injuries, after the copious evidence already adduced in its favour, the following cases, which fell under our own observation, may not, perhaps, be deemed wholly unimportant.

Ann Dawson, a young woman, during the summer of the year 1787, fell into a cistern of boiling water; in consequence of which, her legs, thighs, back, loins, and abdomen were so dreadfully scalded, as to be nearly deprived of the whole cuticle. Cloths wetted with the aqua lythargari acetati composita were kept constantly applied to the injred parts, by which the pain and irritation were so much relieved, within the space of four or five hours, that she fell asleep. As, however, the patient was often roused from her slumbers by the recurrence of pain, it was found necessary to continue the refrigerant treatment for a few hours longer, when complete ease was obtained. The excoriated parts were dressed for some days with unguentum simplex, but afterwards, on the suppuration becoming copious, with the ceratum e lapide calaminari, and other desiccative applications, by which means the cure was completed in the course of seven weeks from the occurrence of the accident.

Except a single opiate given immediately after the infliction of the injury, and one or two laxatives during her convalescence, no other medicine was administered.

Edward Hill, by trade a Carpenter, during 1790, as he was incautiously melting some pitch, it caught fire, which communicating to his clothes, set them in a blaze. Fortunately, he did not lose his presence of mind, for finding his efforts unavailing to extinguish the flame, he immediately plunged into a pool close by the house where the accident happened, and thus escaped inevitable destruction. His face, neck, and head suffered considerably, but the parts most severely injured were his hands and arms, the cuticle of which was not only destroyed, but the cutis vera much burnt and contracted in many places. Linen cloths soaked in a solution of the acetite of lead were applied to the parts, and afforded him perfect relief in the course of a few hours. The injured parts were then dressed with a liniment of oil and wax; but as the suppuratory process proceeded very tardily, warm digestives, and emollient poultices, were afterwards applied, on which the injured and disorganised cutis soon sloughed off, and the sores healed rapidly.

W. F. a boy, about three years old, had the misfortune, while labouring under the eruptive fever of the small-pox, to have his arm scalded with boiling water from the elbow to the extremities of the fingers. The injured arm was instantly immersed in a vessel of cold water, which was ordered to be renewed occasionally as it acquired heat. So sensible was the child of the benefit resulting from it, that whenever he was disturbed by the recurrence of pain during the night, he called for a fresh supply; which was no sooner furnished than he immediately again fell asleep. On the following morning, the arm exhibited no other sign of having been injured than a slight superficial redness, which disappeared in the course of a few days.

Mr.

Mr. Wm. Hilson, an intelligent manufacturer, in the town of Jedburgh, had, in the year 1797, one leg and foot severely scalded with boiling water. He immediately plunged it into a neighbouring stream, and kept it immersed therein for about half an hour. At the termination of this period, finding the pain considerably abated, and wishing to obtain advice respecting its subsequent treatment, he walked to my house, which was situated at the distance of a quarter of a mile from the place where the accident happened. As the pain had recurred during this short walk, he was enjoined to immerse the injured part in a bucket of cold spring water, and renew it occasionally. After the space of three or four hours, he remained wholly exempt from pain, and experienced no other inconvenience than one or two slight vesications, which soon healed, and did not prevent him from resuming his accustomed avocations on the following day.

A. H. a young lady, during the winter of 1802, had one of her legs and feet dreadfully scalded, by having the whole contents of a large tea kettle of boiling water spilt upon them. By incautiously removing the stocking, the cuticle was stript off from all the inferior part of the leg, the whole of the ancle, as well as from the upper part, and sides of the foot. The injured limb was as quickly as possible plunged into a vessel of cold water, which was not only frequently changed, but even rendered still colder by the addition of snow and ice. This in a short time so far relieved the excruciating pain, that the patient evinced a disposition to sleep. She was accordingly put into bed, and the limb placed in such a position, that it was exposed to the external air with no other covering than a thin linen rag wetted in cold water. The relief obtained, was, however, only transient, for in a very short time, the return of pain rendered it again necessary to recur to the former treatment. With this view, she was placed on a couch, in a half recumbent posture, with the limb immersed in cold water, while, at the same time, the nurse and other attendants were enjoined occasionally to change it. In this situation, she continued to sleep profoundly till a late hour in the morning, after which the pain no longer returned.

The subsequent treatment was in no respect different from that usually employed in similar cases. In consequence, however, of the injury sustained by the true skin in this case, and the copious suppuration that ensued, nearly six weeks elapsed before the cure was completed.

We might here mention numerous other cases, in which the employment of topical cold proved equally beneficial; but as the principal features of these cases were the same as those already detailed, it would be superfluous to swell the present communication by their insertion. On the whole, though inclined at present to consider the refrigerant mode of treatment, under certain limitations, as best adapted to the cure of scalds and burns, we yet trust we shall keep our mind open to conviction, and be ready to yield up that judgment, should hereafter the stimulant plan appear to us, on farther investigation, to possess the high merits ascribed to it. *Non enim me cuiquam mancipavi, nullius nomen fero: multum magnorum virorum judicio credo, aliquid et meo vindico.*

I am, &c.

R. HALL.

*Clement's Inn, Jan.* 1, 1807.

